# Supplementary material for: Research on social bot identification through behavioral feature analysis
Source: PLoS One. 2025 Jun 6;20(6):e0324539. doi: 10.1371/journal.pone.0324539 (PMC12143522; doi:10.1371/journal.pone.0324539)
Supplement: Attachment — Submitted filename: Revision Notes.docx [file pone.0324539.s002.docx]

Dear reviewers,

Thank you for your valuable review comments on our paper.Your detailed suggestions have helped us to identify key parts of the article that need improvement and provided important guidelines for refining the paper.We have thoroughly revised and optimised the article, and the following is a point-by-point response to specific questions:

**Reviewer 1:**

**Q1: Quantify the impact of the proposed method in the abstract.**

**A1:** We have added a quantitative description in the abstract, which clearly lists the specific effects of the proposed method in improving model performance, so that readers can more intuitively understand the actual contribution of the method.

**Q2: The contribution, motivation, and challenges of your dissertation work need to be rewritten clearly.**

**A2:** We have completely rewritten the introduction section to present the research value of the paper more clearly.The motivation of the research work has been elaborated by combining the research background and practical problems in the current field, and the contribution points of the thesis as well as the technical challenges faced have been clarified at the same time.

**Q3: Write a clear literature review section.**

**A3:** We have made the literature review part independent of the second section, and have systematically sorted out and expanded the related studies to more clearly describe the progress of the existing studies and their limitations, so as to highlight the innovations of this paper's work.

**Q4: It is better to attach a workflow diagram/pseudo-code.**

**A4:** In order to improve the intuition of the paper, we have separated the methodology section into Section IV and added a detailed workflow diagram to show the specific steps of the proposed method.

**Q5: At what cost is the superior performance of the proposed methodology achieved?**

**A5:** We have supplemented the model analysis with relevant descriptions.By using the OOB method for feature filtering, the complexity of the features is significantly reduced, and despite a slight decrease in model performance, the overall level remains similar to that when using full features.Meanwhile, the computational complexity of Random Forest is comparable to other mainstream models while the performance is improved.

**Q6: Detailed analyses of the limitations and potential failures of the proposed model are missing.**

**A6:** Thanks for your correction.We have systematically analysed the limitations and potential failure scenarios of the model, such as feature dependency, limitation of the model's generalisation ability, and difficulties in the recognition of highly stealthy robots, in the new Section VI.At the same time, we present a detailed outlook on future improvement directions.

**Q7: More comparative analysis with other SOTA methods around computational requirements and model robustness.**

**A7:** The focus of this paper is to analyse the effectiveness of OOB feature selection methods, mainly focusing on the optimisation capability of feature engineering.The results of feature engineering are general in nature and are equally applicable to SOTA models.A better machine learning model (Random Forest) is chosen here just for the integrity of the testing system.

**Reviewer 2:**

We have made the following optimisation adjustments to the article based on your suggestions:

1. in order to improve the logical structure of the paper, the introduction and literature review are separated into two separate sections, which further clarify the research background and the shortcomings of the existing work, and highlight the innovations of this paper.

2. The methodology and experiments are separated into two sections to describe the proposed methodology and experimental design more systematically and to enhance the logical coherence of the content.

3. The experimental principles have been integrated into the methodology, which makes the content of the paper more compact and conforms to the writing standard of academic papers.

4. The overall language of the paper has been refined and embellished to enhance the clarity and professionalism of the presentation and to ensure that readers can clearly understand the core content and value of the study.

In addition, we have substantially revised the structure of the whole article to make it more in line with the writing structure of a research paper.Since we made changes to most of the article, we did not highlight the changes.

Finally, we would like to thank the reviewers again for their valuable comments on our paper.Your suggestions have greatly contributed to the quality of the paper and provided us with new ideas for our subsequent research.

Yours sincerely,

Peng Zhang
